# Pain Sensitivity in Adolescent Males with Attention-Deficit/Hyperactivity Disorder: Testing for Associations with Conduct Disorder and Callous and Unemotional Traits

**DOI:** 10.1371/journal.pone.0134417

**Published:** 2015-07-30

**Authors:** Clare Northover, Anita Thapar, Kate Langley, Stephanie HM van Goozen

**Affiliations:** 1 School of Psychology, Cardiff University, Cardiff, United Kingdom; 2 MRC Centre for Neuropsychiatric Genetics and Genomics, Cardiff University, Cardiff, United Kingdom; National Center of Neurology and Psychiatry, JAPAN

## Abstract

**Background:**

Reduced processing and experience of aversive emotional cues is a common component of theories on the development and persistence of aggression and antisocial behaviour. Yet physical pain, arguably the most basic aversive cue, has attracted comparatively little attention.

**Methods:**

This study measured pain sensitivity and physiological response to painful stimuli (skin conductance level, SCL) in adolescent boys with Attention-Deficit/Hyperactivity Disorder (ADHD; n = 183), who are at high risk for antisocial behaviour. We compared boys with ADHD with and without a comorbid diagnosis of Conduct Disorder (CD) on pain sensitivity, and examined patterns of association between pain measures, on the one hand, and problem severity and callous and unemotional (CU) traits, on the other.

**Results:**

Boys with comorbid CD exhibited a higher pain threshold and tolerance than boys with ADHD alone, but the groups did not differ in physiology at the time the pain threshold and tolerance were reported. Regression analyses showed that ADHD problem severity positively predicted pain sensitivity, whereas levels of CU traits negatively predicted pain sensitivity.

**Conclusions:**

These findings on physical pain processing extend evidence of impairments in aversive cue processing among those at risk of antisocial behaviour. The study highlights the importance of considering comorbidity and heterogeneity of disorders when developing interventions. The current findings could be used to identify subgroups within those with ADHD who might be less responsive to interventions that use corrective feedback to obtain behaviour change.

## Introduction

Research findings highlight the importance of impaired affective response in children with Conduct Disorder (CD; [1–3]). Specifically, impaired fear conditioning [4–6], attenuated startle and cortisol stress response when emotionally challenged [3, 4, 7], poor recognition of negative facial expressions [8, 9], and fearlessness and insensitivity to punishment [7, 10] suggest reduced experience and processing of aversive emotional cues in those with CD. Physical pain is arguably the most basic and fundamental form of aversive cue, yet very little research has looked at pain sensitivity in children with CD or antisocial behaviour more generally.

Previous research has focused on the relation between pain sensitivity and aggression due to the argument that pain is a trigger to aggression because of its provocative nature [11]. However, acting aggressively towards others has been found to be associated with a high rather than a low pain threshold [12–14], suggesting that pain sensitivity in some people is so low that it no longer acts as a motivational factor to regulate behaviour. Seguin et al. [12] studied pain tolerance and aggression in adolescent boys with a history of aggressive behaviour. Pain tolerance was assessed via finger pressure stimulation and aggressive behaviour was assessed over a 7-year period. The authors found that adolescents who showed persistent aggressive behaviour displayed the highest pain tolerance. However, pain sensitivity might also be associated with personality more generally; for example, reduced pain sensitivity has been found in extroverted and sensation-seeking individuals [15], as well as incarcerated antisocial adults [16].

Children and adolescents with Attention-Deficit Hyperactivity Disorder (ADHD) are at high risk of displaying conduct problems. Approximately 30–50% of those with ADHD also meet criteria for a comorbid diagnosis of CD [17]. Furthermore, when ADHD coexists with antisocial behaviour both problems are clinically more severe and persistent, and have a worse prognosis than when they occur alone [18]. ADHD has been linked to abnormalities in somatosensory processing, which involves the processing of sensations from the body such as tactile and kinaesthetic information [19, 20]. However, few studies have looked at pain perception. Studies in adult samples with ADHD have found higher levels of chronic [21] and widespread pain [22] compared to controls, but no difference in the report of past painful experiences [19]. Treister at al. [23] recently objectively measured pain sensitivity in adult participants with ADHD (n = 30) and controls (n = 30), and found that the ADHD group had a significantly lower pain threshold and tolerance time than the control group. Until now no study has examined pain sensitivity in children with ADHD or looked at the effect of comorbidity within ADHD. The present study examined pain perception in a large sample of adolescent boys with ADHD, and examined the effect of severity of ADHD and CD symptoms on pain sensitivity.

Another variable closely related to conduct problems is psychopathic traits [24]. Psychopathic traits are related to a lack of empathy for others’ pain [25, 26]; one might therefore speculate that individuals, who are less responsive to self-experienced pain, might also have difficulty appreciating others’ pain [27]. Research findings regarding self-experienced pain in psychopathy are mixed [27]. Previous research suggests that individuals high in psychopathic traits take longer to detect the presence of an electric shock, but do not differ in pain tolerance [28], although these individuals may tolerate higher levels of pain in response to incentives [29]. Fedora and Reddon [16] compared pain tolerance to electrical stimulation in prisoners high and low in psychopathic traits and found that both prisoner groups had a higher pain tolerance than a control group, but did not differ from one another. Cheng et al. [30] obtained the same results when comparing high and low callous and unemotional juvenile offenders. These personality traits, it is argued [31], identify those at greater risk for severe antisocial behaviour and reduced responsiveness to treatment [32]. The importance of CU traits has been acknowledged by including limited prosocial emotions as a specifier for CD in the fifth edition of the Diagnostic and Statistical Manual of Mental Disorders [33, 34]. We therefore aimed to explore the effect of CU traits in this sample.

Pain is an extremely difficult outcome to measure due to its subjective nature [35]. Previous research in antisocial groups has mainly used self-report measures of pain sensitivity by either asking participants to report their threshold and/or tolerance points, or by taking self-report ratings of experienced pain (e.g. [36, 37]). These types of measurements, however, can be influenced by reporter biases, especially the motivation to impress the experimenter [38]. Brown, Sheffield, Leary and Robinson [39] showed that the mere presence of another person affected pain tolerance, and Reidy, Martinez and Zeichner [13] found that pain tolerance was correlated with participants’ conformity to gender roles. It would be interesting to measure physiological responses to pain to analyse the relationship between these and self-report measures. Autonomic nervous system responses are related to aversive stimuli and reduced in antisocial samples [40]. Measurements of changes in skin conductance level have been used in pain research and found to correlate with self-reported ratings of pain (e.g. [41, 42]). However, until now no study has examined the association between subjective and physiological responses to pain in high risk, clinically antisocial populations.

In this study we hypothesised that adolescents with ADHD and comorbid CD would have lower pain sensitivity (i.e., higher threshold and tolerance to pain) than those with ADHD alone, and that CD symptom severity and/or CU traits would inversely predict pain sensitivity.

## Methods

### Sample

Participants were recruited from Child and Adolescent Mental Health Services and Community Child Health Clinics in Wales. Children in the sample were of British Caucasian origin and met research criteria for a lifetime DSM-IV diagnosis of ADHD. Children with any known clinical or research diagnosis of schizophrenia, bipolar disorder, Autistic Spectrum Disorder (ASD), Tourette’s syndrome, or with an IQ<70 (based on the administration of the Wechsler Intelligence Scale for Children, WISC [43]), epilepsy, brain damage or any other neurological or genetic disorder were excluded from the study. In total 204 adolescent males with ADHD (mean age = 13.95 years, sd = 1.82; age range 10–17 years) took part. All participants came from community clinics and none were stimulant naive. Participants who continued to take ADHD medication (74.2%) were asked to come off medication at least 24 hours prior to testing.

### Ethics Statement

Approval for this study was obtained from the South Wales Multicentre Research Ethics Committee. Informed written consent was obtained from parents of all participants and from adolescents aged over 16 years. For younger adolescents, written assent was obtained (in addition to the written consent from parents).

### Clinical Measures

Child psychopathology was assessed using the Development and Well Being Assessment (DAWBA) structured interview using parents and children as informants [44]. Parents completed the ADHD and CD sections and children the CD section. All interviews were administered by trained psychologists, supervised by an experienced clinician (AT). ADHD and CD diagnoses and symptom scores were generated from the DAWBA according to DSM-IV criteria (3+ symptoms; DSM-IV was still in use at the start of the study). CD symptoms were counted as present when endorsed by either the parent or child. Based on this information participants were subdivided into two groups: ADHD only or ADHD with a research diagnosis of CD (ADHD+CD).

Callous-Unemotional traits were measured using the Youth Psychopathic traits Inventory (YPI; [45]). The CU subscale has 15 items, and each item is answered on a 4-point Likert scale (score range 0–45). These 15 items were summed to achieve a CU trait score (‘CU’). The reliability and validity of the YPI have been established [46].

Self-rated emotional/anxiety symptoms were assessed using the Strengths and Difficulties Questionnaire (SDQ; [47]) completed as part of the DAWBA. The five emotional items were summed to obtain a total emotional symptom score (‘SDQ emotion’).

Cognitive ability was assessed using the Wechsler Abbreviated Scale of Intelligence [48] – 2-subset form (vocabulary and matrix reasoning) to create an Intelligence Quotient variable (‘IQ’).

### Procedure

The pain induction procedure replicated the procedure described by Thompson, Keogh, French and Davis [49]. A Peltier-based thermode with a 5 cm by 5 cm aluminium contact pad was used for the thermal heat induction (www.psyal.co.uk). Participants were first asked to do a practice trial to familiarise themselves with the heat sensation and eliminate any substantial differences in baseline hand temperatures. The experimenter positioned the thenar eminence of the participant’s non-dominant hand on the contact pad and participants were asked to hold it there for 30 seconds, with the temperature set at 40°C. For the experimental trial, the temperature of the pad was increased to 48°C. Participants were asked to report when the heat sensation began to elicit pain (‘threshold time’) and when the pain became too much, at which moment they stopped the procedure by removing their hand from the thermode (‘tolerance time’). These two self-reported times were recorded in seconds and participants were asked to stop after 90 seconds if they had not already done so. This limit was established as appropriate during pilot testing.

### Skin Conductance Recording

Electrodermal activity was recorded using a skin conductance amplifier (PSYCHLAB Contact Precision Instruments, UK). Skin conductance paste (ABRALYT 2000, Chloride free abrasive electrolyte gel, supplied by Falk Minow Services DE-82211 Herrsching) was used to fill the 8 mm diameter silver/silver chloride electrodes, which were placed on the distal phalanges of the index and middle fingers of the dominant hand, using double-sided adhesive electrode collars.

SCL at reported threshold time (‘SCL_threshold_’) and tolerance time (‘SCL_tolerance_’) were recorded. In order to confirm that the SCL recording related to participant’s perception of pain, the increase in SCL from the start of the procedure to the termination point was used to predict tolerance time. A larger increase in SCL was expected to predict a shorter tolerance time. The individual’s increase in SCL was then divided by their tolerance time to create a pain ratio variable (‘pain ratio’). Because participants would vary in how long their hands were on the pain stimulus it was important to take this into account when looking at their SCL during this time. A high pain ratio reflects a rapid SCL increase over a short tolerance time, signifying high sensitivity to pain, whereas a low pain ratio value reflects a slow SCL increase over a protracted tolerance time, reflecting low sensitivity to pain.

#### Data analysis

Two participants were excluded because of missing or incomplete DAWBA data, and eighteen participants had missing pain data, resulting in a sample of 183 participants. Over half (53.3%) of these met criteria for a comorbid diagnosis of CD. This is at the high end of estimated rates in ADHD [17]. Prevalence rates reported in the literature are usually based on younger age groups and our sample included adolescents (mean age = 13.97), with CD increasing with age. Reported and SCL threshold and tolerance variables were not normally distributed and therefore transformed using a log10 transformation. Transformation led to the SCL variables becoming normally distributed and the self-report variables becoming less skewed, but these were still not normal. However, comparison of parametric and non-parametric Mann-Whitney U test results led to the same findings. This outcome in combination with the large sample size gave us the confidence to use parametric tests; this also enabled us to carry out further analyses and look at the covariance effect of IQ. Between group differences were assessed using ANOVAs. Effect sizes are reported as eta squared (η^2^_*p*_; small≥.01, medium≥.06, large≥.14; [50]). Finally, Pearson correlations and multiple regressions examined the effect of clinical and demographic characteristics on pain variables and SCL. Analyses were carried out using SPSS 16.0 (SPSS Inc., Chicago, IL). The dataset underlying the reported findings is available in S1 Dataset.

## Results

### Methodology check

The increase in SCL from baseline to pain tolerance point significantly (inversely) predicted pain tolerance time (*F*[1, 183] = 34.1, *p* < .001, *R* = .40, Beta = -.40) suggesting that those with a slower SCL increase were able to keep their hand longer on the thermode. We then divided the SCL by tolerance time to create the pain ratio; this also significantly predicted tolerance time (*F*[1, 183] = 41.7, *p* < .001, *R* = .43 Beta = -.43). Because these two variables were highly correlated (r = .72), we subsequently only used the pain ratio in further analyses.

### Clinical groups

The demographic data for the two subgroups and the results of the between-group analyses are presented in Table 1.

**Table 1:** Demographic and clinical characteristics of the ADHD and ADHD+CD subgroups.

	ADHD (N = 85)		ADHD+CD (N = 98)		
	*Mean*	*SD*	*Mean*	*SD*	*t-value*
Age (years)	13.85	1.86	14.09	1.71	ns
IQ	90.26	9.86	84.76	9.33	*p* < .001
ADHD symptoms	11.67	4.86	13.17	4.27	*p* < .05
CD symptoms	1.00	0.80	5.77	2.42	*p* < .001
CU	16.39	5.96	20.77	2.01	*p* < .001
SDQ emotion	4.07	2.37	3.82	2.36	ns

Note: ADHD symptoms = ADHD symptom score; CD symptoms = CD symptom score; CU = callous/unemotional trait score; SDQ emotion = Strengths and Difficulties emotional symptom subscale score.

With respect to subjectively reported pain, the ADHD+CD group had a higher pain threshold time (M = 21.25, SE = 2.74) than the ADHD only group (M = 14.71, SE = 2.54; *F*[1, 181] = 5.19, *p* = .024, η^2^_*p*_ = .03). The ADHD+CD group also had a higher tolerance time (ADHD+CD: M = 40.60, SE = 3.07; ADHD: M = 30.38, SE = 3.24; *F*[1,181] = 8.04, *p* = .005, η^2^_*p*_ = .04). When we controlled for the group differences in IQ and ADHD symptom scores the difference in threshold time (*F*[1, 172] = 5.96, *p* = .016) and tolerance time (*F*[1, 172] = 9.85, *p* = .002) remained significant. Results of non-parametric Mann-Whitney U tests comparing ADHD and ADHD+CD groups revealed the same pattern of results: Threshold time: *U* = 3328, *p* = .019; Tolerance time: *U* = 3216, *p* = .008; SCL_threshold_: *U* = 3898.5, *p* = .456; SCL_tolerance_: *U* = 3750.5, *p* = .246.

**Fig 1:**
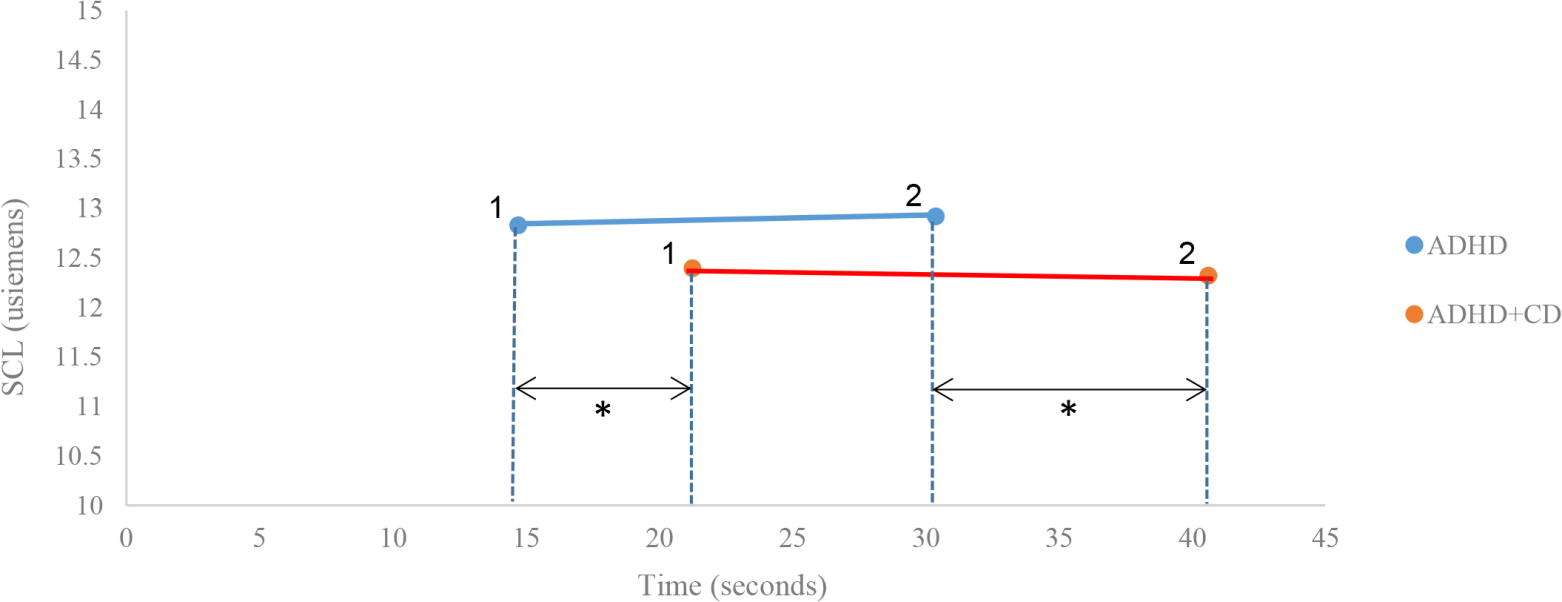
Mean skin conductance level at threshold and tolerance time for the ADHD and ADHD+CD groups. 1 = threshold time; 2 = tolerance time; * = *p* < .05.

There were no differences between the ADHD and ADHD+CD groups in SCL_threshold_ (*F*[1, 181] = .78, *p* = .38, η^2^_*p*_ = .004), or SCL_tolerance_ (*F*[1,181] = 1.35, *p* = .25, η^2^_*p*_ = .007), nor was there a difference in pain ratio (ADHD: M = .08, SD = .13; ADHD+CD: M = .07 = .18; *F*[1,181] = .04, *p* = .84). Fig 1 illustrates that although the groups differed in pain threshold and tolerance, there were no differences in the physiological response at these points in time. This means that both groups had the same SCL when they reached their threshold and tolerance points, but that the ADHD+CD group took significantly longer to get there.

There was no difference between ADHD participants who did and did not take medication on any of the pain variables (Threshold time: *p* = .50; Tolerance time; *p* = .21; SCL_threshold_: *p* = .34; SCL_tolerance_: *p* = .31; SCL_increase_: *p* = .70; Pain ratio: *p* = .77).

### Clinical measures

Table 2 shows the pattern of correlations between the clinical variables and the pain measures.

**Table 2:** Pearson’s correlations between clinical and pain sensitivity measures.

	ADHD	CD	CU	SDQ emotion	Threshold time	Tolerance time	SCL_threshold_	SCL_tolerance_	Pain ratio
ADHD	-								
CD	.19**	-							
CU	.11	.40**	-						
SDQ emotion	-.10	-.05	-.05	-					
Threshold time	-.18*	.17*	.12	-.04	-				
Tolerance time	-.14	.19*	.21**	.10	.68**	-			
SCL_threshold_	.06	-.07	-.04	.14	-.03	-.06	-		
SCL_tolerance_	.09	-.07	-.05	.10	-.10	-.11	.98**	-	
Pain ratio	.17*	-.01	-.17*	-.04	-.31**	-.50**	.36**	.43**	-

Note

* = p < .05

** = p < .001. ADHD = ADHD symptom score; CD = CD symptom score; CU = callous/unemotional trait score; SDQ emotion = Strengths and Difficulties emotional symptom subscale score; Threshold time = time at which first experience of pain is reported; Tolerance time = length of time until procedure is terminate; SCL_threshold_ = Skin conductance level at threshold time; SCL_tolerance_ = Skin conductance level at tolerance time; Pain ratio = Skin Conductance increase divided by tolerance time.

Table 2 shows that CD and ADHD symptom scores were each significantly correlated with pain threshold time. A multiple regression confirmed that both ADHD and CD symptom scores significantly predicted pain threshold time (*F*[2, 175] = 7.26, *p* < .001, *R* = 0.28); however, ADHD symptom scores inversely predicted pain threshold time (Beta = -0.23) and CD symptom scores positively predicted threshold time (Beta = 0.21).

CD symptom scores and CU traits were highly correlated, and each correlated significantly with pain tolerance time. A stepwise regression revealed that only CU traits significantly predicted pain tolerance time (*F*[1, 181] = 8.37, *p* = .002, *R* = 0.21, Beta = 0.21), with CD symptom score not adding to the model (*p* = 0.16).

ADHD symptom scores and CU traits both correlated significantly with the pain ratio reflecting physiological sensitivity to pain. A multiple regression showed that both ADHD symptom scores and CU traits significantly and independently predicted the pain ratio (*F*[2, 171] = 6.24, *p* = .002, R = 0.26), with ADHD positively (Beta = 0.20) and CU traits negatively predicting it (Beta = -0.20). Total CD scores did not predict the pain ratio.

## Discussion

This was the first study to measure the effects of comorbid CD and CU traits on pain sensitivity in a large sample of adolescent boys with ADHD. Furthermore, no previous studies on pain in children with behavioural difficulties measured the physiological response to pain. SCL was measured during the procedure to investigate whether self-reported threshold and tolerance times were associated with participants’ physiological responses to pain. We found that the increase in SCL from the start of the pain procedure to participants’ tolerance time significantly predicted tolerance time, suggesting a larger physiological response reflected a greater experience of pain. This provides support for using SCL as a measure of pain sensitivity alongside self-report and behavioural measures in these samples.

We found that males with ADHD and comorbid CD took longer to report initial pain and were able to endure it for longer, as reflected in their significantly higher pain threshold and pain tolerance times than those with ADHD only. A relatively high pain threshold is thought to reflect a lower sensitivity to aversive stimulation, which has previously been found in adolescents with CD (e.g. [3, 10]). If negative stimuli are experienced as less aversive and consequently have less punishing effects, then it may be more difficult to socialize these individuals to behave in a prosocial manner [51]. However, although the groups differed in pain behaviour, there were no differences in the physiological response to pain at these points in time. This means that although both groups had the same SCL when they reached their threshold and tolerance points, the ADHD+CD group took significantly longer to get there (see Fig 1). When examining relations with the clinical variables we found that CU traits predicted low physiological response to pain, whereas ADHD severity predicted higher physiological response. Specifically, more severe ADHD was associated with a more rapid SCL increase over a short tolerance time, whereas higher levels of CU traits were associated with a slower SCL increase over a protracted tolerance time.

Research shows that pain is perceived as less intense when individuals are distracted [52]. Spontaneous hypertensive rats (SHR; a widely used animal model of ADHD; [53]) have been found to show reduced pain sensitivity, yet their pain receptor neurons appear to be normal. In a study in which rats were first habituated to a hot plate the SHR rats no longer showed reduced pain sensitivity [54]. This suggests that the pain insensitive phenotype of these SHR rats involves cognitive processes, for example, distraction. These findings suggest ADHD would be associated with a higher pain threshold. However, Treister at al. [23] found that those with ADHD had a significantly lower pain threshold and tolerance time than controls and hypothesised that this was due to dopamine dysregulation. There is growing evidence that dopamine dysregulation plays a role in the neurobiology of ADHD [55] and in the processing of pain [56]. Our results in an adolescent sample with ADHD support this as we found ADHD severity was associated with a greater sensitivity to pain.

A growing number of functional magnetic resonance imaging (fMRI) studies have shown remarkable similarities in the neural circuits involved in the processing of own and others pain [57–61]. The results from the present study add to this debate: participants high in CU traits, who arguable lack empathy, showed an increased tolerance and slower physiological response towards their own pain, which might explain why they also have less empathy for others’ distress.

The study had some limitations. First, we examined pain processing within a large sample of clinical cases with ADHD and there was no normal healthy control group for comparison. Only if a normal control group is included can we establish that ADHD is associated with higher or lower pain sensitivity. Second, future research could also include a sample of participants with CD without ADHD. If ADHD is associated with a lower and CD with a higher pain threshold then individuals with CD without comorbid ADHD might have an even higher pain threshold than the participants in this study, and this would be an important observation. Third, we can not be certain that the increase in SCL reflects the intensity of physical pain rather than other emotions (e.g. fear or excitement), since SCL changes are valence nonspecific. It is possible that the ADHD+CD group felt more ambivalent towards the pain stimulus or enjoyed it more and therefore had a greater pain tolerance. It may be beneficial in future research to measure other self-reported emotions. Furthermore, the accuracy of measuring the physiological response to pain could be improved by including other measures, for example, pupil dilation or heart rate [41, 42].

Lastly, we were unable to analyse in more detail the effect of medication on pain perception. Treister et al. [23] found that the psychostimulant drug methylphenidate (Ritalin) increased pain threshold and tolerance in adults with ADHD. Although we asked our participants to come off medication 24 hours before testing, psychostimulant medication varies in how long it takes to leave the body. However, we found no differences on any of the pain variables between those with a prescription for medication and those without. Future research should, however, look into the different anti-nociceptive effects of different medications.

This study highlights the importance of considering comorbidity and heterogeneity of disorders when developing interventions. If reduced pain sensitivity reflects reduced reactivity to aversive cues more generally, including punishment, then punishment-based interventions for troublesome behaviour are less likely to be effective in treating certain types of CD, particularly those high in CU traits. Conversely, if ADHD without CD is associated with an increased aversive cue sensitivity, interventions involving corrective feedback and learning from punishment are a treatment option for those with this behavioural profile.

There is very little research on the role of pain sensitivity in development more generally and psychopathology more specifically. We do, for example, not know whether pain sensitivity is a precursor of antisocial development. A young child with a high pain threshold might be less reserved about engaging in risky behaviour and less responsive to corrective feedback [62]. We also do not know much about the stability of, or individual differences in pain sensitivity. It is possible that pain thresholds change as a result of exposure to external events, for example in the case of a ‘toughening up’ as a result of childhood adversity, whilst being exposed to harsh parental discipline, childhood abuse or peer victimization. Longitudinal research is clearly needed to shed light on these important issues.

## Supporting Information

S1 Dataset SPSS.xls file containing dataset underlying reported findings. (XLSX)
